# Increased Density of the Liver and Amiodarone-Associated Phospholipidosis

**DOI:** 10.4061/2009/598940

**Published:** 2009-09-13

**Authors:** Sunao Kojima, Shinobu Kojima, Hirofumi Ueno, Motohiro Takeya, Hisao Ogawa

**Affiliations:** ^1^Department of Cardiovascular Medicine, Graduate School of Medical Sciences, Kumamoto University, Kumamoto 860-8556, Japan; ^2^The Second Department of Pathology, Graduate School of Medical Sciences, Kumamoto University, Kumamoto 860-8556, Japan

## Abstract

This is a case report in which a 60-year-old man who suffered from ventricular tachycardia with dilated cardiomyopathy was prescribed amiodarone. After taking amiodarone, liver enzymes were increased and computed tomographic (CT) scanning of the abdomen showed a significant increase in the density of the liver without contrast medium. He was suspected as hemochromatosis and liver biopsy was performed. An abnormal high density of liver tissue may be observed in an unenhanced CT in patients treated with amiodarone and we suggest that periodic monitoring of liver function and/or liver biopsy is warranted before an irreversible stage is reached.

## 1. Introduction

Amiodarone is an antiarrhythmic drug used in patients with refractory supraventricular and ventricular tachycardia and is available worldwide. Pulmonary fibrosis is the most well-known complication of amiodarone. Abnormal liver function is also well known, however, that long-term treatment with amiodarone increases computed tomography (CT) density of the liver by the iodinated drug/metabolites has not been reported recently. In this paper, we describe an abnormal high density of liver tissue in an unenhanced CT in a patient treated with amiodarone and a great consequence of periodic monitoring of liver function and/or liver biopsy.

## 2. Case Report

**Figure 1:**
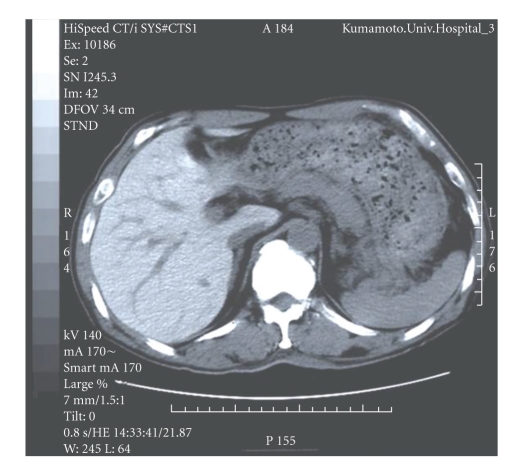
Abdominal computed tomographic scanning through liver and spleen. Note the marked increase in liver density (113 Hounsfield units) compared to the spleen.

**Figure 2:**
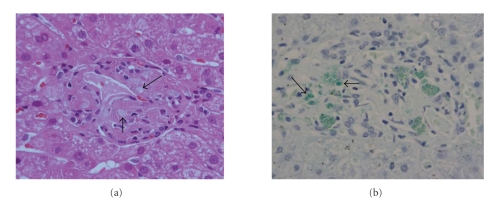
Photomicrographs of a liver biopsy. (a) Macrophages infiltrate into the portal area and contain achromatic fine granular depositions (arrows, hematoxylin, and eosin staining). (b) Fine granules are stained green in Leuco malachite green staining (arrows). Magnification, ×400.

**Figure 3:**
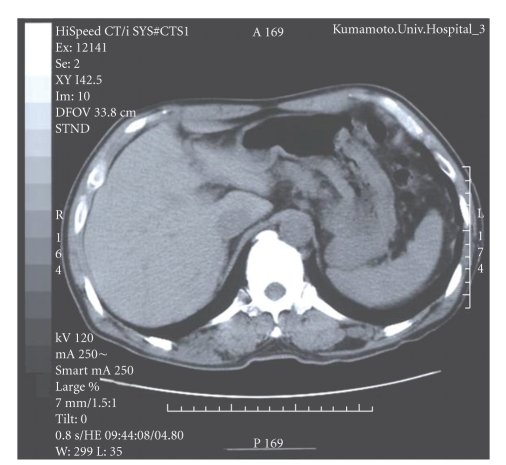
Normalization of CT density of the liver after 6-month discontinuation of amiodarone (63 Hounsfield units).

A 60-year-old man who suffered from ventricular tachycardia with dilated cardiomyopathy was prescribed 400 mg amiodarone daily. About 18 months after taking amiodarone, liver enzymes were noted to have increased and computed tomographic (CT) scanning of the abdomen showed a significant increase in the density of the liver without contrast medium, compared to that of the spleen and surrounding tissues (113 Hounsfield units (H), normal range of the liver is about 30–70 H, Figure 1). Primary hemochromatosis was suspected. A liver biopsy was performed under direct laparoscopic visualization. However, he developed complete atrioventricular block immediately after the procedure and was transferred to Kumamoto University Hospital for urgent insertion of a temporary pacing lead. Amiodarone blood concentration was 1554 ng/mL and the level of its active metabolite desethylamiodarone was 1456 ng/mL. Hematoxylin and eosin staining of the liver biopsy revealed mild expansion of the portal area without intrinsic hepatocellular disease. No evidence of abnormal hemosiderin deposition was observed in Berlin blue staining. The striking feature, however, was fine granular depositions in the macrophages that accumulated in the portal area (Figure 2(a)). These fine granules showed distinct staining patterns in various staining methods as follows: periodic acid Schiff: purple red, Ziehl-Neelsen: dark red, Sudan Black B: black, Nile blue: blue, Schmorl: light blue (data not shown), and Leuco malachite green: green (Figure 2(b)). Based on these staining patterns, the fine granular depositions were considered to be lipopigments (ceroid and lipofuscin). Marked fibrosis and bridging necrosis within the portal spaces consistent with liver cirrhosis were not observed. At about 3 months after withdrawal of amiodarone, the liver enzymes returned to normal levels and the liver density became normal (63 H, Figure 3). Furthermore, amiodarone and desethylamiodarone became undetectable in the blood at about 6 months after cessation of amiodarone.

## 3. Discussion

Amiodarone and desethylamiodarone are likely to accumulate in the liver rather than skeletal muscle and spleen [1]. Furthermore, the levels of amiodarone and desethylamiodarone correlate with liver CT density [2]. Amiodarone (2-butyl-3-benzofuranyl 4-[2-(diethylamino) ethoxy]-3, 5-diiodophenyl ketone hydrochloride) contains two atoms of iodine, which account for about 37% of the molecular weight of the drug. The accumulation of amiodarone and desethylamiodarone leads to increased iodine content within the liver. Hence, the increased hepatic CT density is probably due to the high concentration of the iodinated drug and its metabolite.

The staining pattern of granular depositions is similar to that of neuronal ceroid lipofuscinosis, which is characterized by accumulation of lysosomal autofluorescent lipopigments (ceroid and lipofuscin) resulting in part from the reduced phospholipase activity [3]. Although the precise mechanism is not elucidated, amiodarone accumulates in lysosomes of macrophages and interacts with phospholipids to form drug-lipid complexes, which seem to be protected from enzymatic attack by phospholipase, leading to a secondary phospholipidosis. This amiodarone-associated phospholipidosis may be observed as fine granular depositions in the macrophages of liver biopsy.

Hepatic enzymes and CT density of the liver normalized after cessation of amiodarone in the present case. Several clinical reports about abnormal high density of the liver in patients treated with amiodarone have been published. However, reports similar to the present case demonstrating liver biopsy as well as periodic monitoring of liver function, blood amiodarone levels and CT density of the liver after cessation of amiodarone are limited. Hepatic function is in general reversible if amiodarone is withdrawn in the early phase. However, hepatotoxicity is considered by amiodarone-induced inhibition of mitochondrial *β*-oxidation and subsequent production of reactive oxygen species. These series may cause apoptosis and necrosis of hepatocytes, leading to nonalcoholic steatohepatitis [4]. In fact, a previous case report showed that long-term administration of amiodarone induced liver cirrhosis [5].

Amiodarone is a clinically beneficial drug in refractory cases of supraventricular and ventricular arrhythmias. However, an abnormal high density of liver tissue may be observed in an unenhanced CT in patients treated with amiodarone and we suggest that periodic monitoring of liver function and/or liver biopsy is warranted before an irreversible stage is reached.

## Abbreviations

CT: Computed tomography
H: Hounsfield units
